# Herbal Medicine in Three Different Mediterranean Living Areas During the COVID-19 Pandemic: The Role of Polyphenolic-Rich Thyme-like Plants

**DOI:** 10.3390/plants13233340

**Published:** 2024-11-28

**Authors:** Mohamad Khalil, Hala Abdallah, Maria Calasso, Nour Khalil, Ahmad Daher, Jihen Missaoui, Farah Diab, Lama Zeaiter, Laura Vergani, Agostino Di Ciaula, Piero Portincasa

**Affiliations:** 1 Clinica Medica “A. Murri”, Department of Precision and Regenerative Medicine and Ionian Area (DiMePre-J), University of Bari Medical School, 70124 Bari, Italy; halaabdallah18@gmail.com (H.A.); agodiciaula@gmail.com (A.D.C.); 2 Department of Soil, Plant and Food Sciences, University of Bari Aldo Moro, Via Amendola 165/a, 70126 Bari, Italy; maria.calasso@uniba.it; 3 Rammal Laboratory, Faculty of Sciences, Lebanese University, Al-Hadath Campus, Beirut 1003, Lebanon; noorkhalil2498@gmail.com (N.K.); ahmad.daher@ul.edu.lb (A.D.); 4 Research Laboratory of BIORESSOURCES—Integrative Biology & Valorisation BIOLIVAL (LR14 ES06) at ISBM, Monastir 5000, Tunisia; missaouijihen@outlook.fr; 5 Department of Earth, Environment and Life Sciences (DISTAV), University of Genova, Corso Europa 26, 16132 Genova, Italy; diabfarah2@gmail.com (F.D.); lama.zeaiter777@hotmail.com (L.Z.); lara.vergani@unige.it (L.V.)

**Keywords:** COVID-19, medicinal plants, Za’atar, post-COVID-19, Mediterranean

## Abstract

Despite herbal medicine being popular across the Mediterranean basin, there is no evidence in favor of COVID-19 infection. This study investigates the utilization and effects of medicinal plants in Italy, Lebanon, and Tunisia during COVID-19 and its effects on post-COVID-19 pandemics. We used a tailored, web-based “Google Form” questionnaire with the random sampling method. We gathered 812 complete responses (Italy: 116, Lebanon: 557, and Tunisia: 139), revealing diverse demographics and symptom experiences. Fatigue prevailed across all groups (89.0–94.2%), while psychological impacts ranged from 20.1% to 30.9%, with higher rates in Lebanon. Post-COVID-19 symptoms affected 22.4% (Italy), 48.8% (Lebanon), and 31.7% (Tunisia). General use of herbs was consistent (41.4–50.4%), with 23.3% (Italy), 50.2% (Lebanon), and 65.5% (Tunisia) employing herbs for COVID-19 therapy. Notably, in Lebanon, Za’atar, a thyme-like plant, correlated with reduced symptoms, suggesting potential protective effects that are likely due to its polyphenol richness. This study underscores the persistent reliance on traditional medicinal plants remedies in the Mediterranean area, with regional variations. Further exploration of herbal compounds for COVID-19-like symptoms is warranted.

## 1. Introduction

The World Health Organization (WHO) defines traditional medicine, including medicinal plants, as having a long history. It is the total of the knowledge, skills, and practices used in the maintenance of health, as well as in the prevention, diagnosis, or treatment of physical and mental disorders [1]. Natural products from medicinal plants and their structural analogs have historically played a significant role in drug development, particularly in treating cancer and infectious diseases [2]. For centuries, natural compounds have been foundational in medicine, with classic examples including digitalis (derived from foxglove), ergotamine (from contaminated rye), quinine (from cinchona bark), and salicylates (from willow bark) [3]. Several prescribed and clinical medicines are originally derived from medicinal plants; however, discovering drugs from natural sources requires a complex approach, integrating botanical, phytochemical, biological, and molecular methods.

A panel of preclinical studies including in vitro cellular and in vivo animal studies, as well as human clinical trials, showed the efficacy of some medicinal plants and bioactive compounds for several diseases including metabolic, neoplastic, neurodegenerative, and infectious diseases [4,5,6,7]. Several medicinal plants are practiced worldwide for respiratory diseases such as obstructive lung diseases [8] and different infectious respiratory diseases [9]. Specific plants, like *Echinacea purpurea* and *Zingiber officinale*, show potential as supportive treatments for respiratory symptoms. Certain plant leaves, like those of *Acacia torta* and *Ocimum sanctum*, are commonly used for ailments like pneumonia and bronchitis [10].

The Mediterranean basin differs from other regions in its rich inventory of herbal medicinal products. The mild climate and the biogeography, geology, and ecological features make the Mediterranean basin unique in terms of biodiversity and plant species with medicinal potential [11]. The use of herbal medicine in the Mediterranean area is an integral part of folk culture, where plants and herbs are largely used for the treatment and/or prevention of a wide spectrum of diseases, including respiratory diseases.

Medicinal plants have long been utilized to treat various infectious diseases, with approximately 25% of commonly prescribed drugs containing plant-derived compounds [12]. Several plants have demonstrated potential in managing viral infections, and advances in vector-based drug discovery and the separation of bioactive compounds represent promising avenues for new drug development [13].

In late 2019, a new strain of coronavirus appeared and was named acute respiratory syndrome coronavirus 2 (SARS-CoV-2) by the International Committee on Taxonomy of Viruses (ICTV) [14] when a cluster of patients with pneumonia of unknown cause was recognized in Wuhan, China [7]. SARS-CoV-2 was the cause of the Coronavirus Disease-19 (COVID-19), which has rapidly increased worldwide due to high transmission rate and severe effects on human health. Since then, around 460 million confirmed cases and 6 million deaths have occurred (“WHO Coronavirus (COVID-19) Dashboard|WHO Coronavirus (COVID-19) Dashboard With Vaccination Data”, n.d.) [15,16,17]. On 11 March 2020, the World Health Organization (WHO) defined COVID-19 as a global pandemic and public health emergency of international concern [5].

Patients infected with SARS-CoV-2 show different clinical manifestations, ranging from an asymptomatic disease to a mild, severe, or fatal illness [18]. The most common symptoms of COVID-19 are fever, cough, and myalgia. Other minor symptoms include sore throat, headache, chills, nausea or vomiting, diarrhea, and conjunctival congestion [6]. Gastrointestinal and hepatobiliary manifestations are also possible in patients with COVID-19 [15,16,19]. Several studies have shown that this coronavirus can also cause long-term complications, as it may cause major injuries to the heart, kidneys, gastrointestinal tract, brain, and even blood vessels [20]. Besides vaccination, various therapeutic approaches for COVID-19 are being employed for both hospitalized adults and children, as well as for outpatients at risk of severe disease [21]. Current treatments include antiviral agents, immunomodulators, and supportive therapies aimed at managing symptoms and preventing complications. However, limitations persist, such as the incomplete understanding of COVID-19’s diverse mechanisms of action and the variability in treatment efficacy across different patient populations. The ongoing emergence of new variants and the potential for long-term sequelae further highlight gaps in therapeutic knowledge, underscoring the need for continued research into optimizing COVID-19 therapies [22].

Numerous phytochemicals with antiviral properties, derived from medicinal plants, are under investigation as potential therapeutic agents for COVID-19 [23,24,25]. Given the experiences from past epidemics, researchers suggest that herbal medicines offer advantages such as lower side effects, affordability, and a reduced likelihood of resistance development. Traditional medicine has also highlighted the effectiveness of essential oils from plants in managing viral respiratory infections [26]. Multiple studies evaluated the efficacy and possible applications of phytomedicine against SARS-CoV-2 [1,27,28]. Research on plant-derived compounds showed that they inhibit virus replication, block viral entry, and modulate immune responses, including ACE-2 receptor and TMPRSS2 blockade, inflammation regulation, and TLR/Nrf2 pathway activation. Notable plants with anti-SARS-CoV-2 potential include *Allium sativum*, *Nigella sativa*, *Glycyrrhiza glabra*, and *Withania somnifera*, while compounds like kaempferol, quercetin, and glycyrrhizin show promise [29]. However, the potential preventive effects of medicinal herbs and their impacts on disease severity following SARS-CoV-2 infection remain largely unexplored and warrant further clinical and epidemiological investigation.

Za’atar is a full expression of the popular plant-based medicine and food in Lebanon, Eastern Mediterranean, and North African countries. As a word, the meanings of Za’atar may differ between regions and cultures. Generally, Za’atar refers to a type of plant, and at the same time to a combination of different plants and spices blended altogether [30,31].

One of the most common Za’atar species is *Origanum syriacum* L., which is widely cultivated across the Mediterranean, Western Asia, and Southern Europe, where it holds significant economic importance [32]. Commonly known as Lebanese oregano, “Za’atar”, Bible hyssop, or Syrian oregano. Additionally, Za’atar is enjoyed as an herbal tea and used as a seasoning in cooking. It is available in several commercial forms, including fresh and dried leaves, as well as essential oil. Particularly in Lebanon and Tunisia, Za’atar refers to many plants belonging to different families called thyme-like plants, including *Origanum*, *Thymus*, *Thymbra*, and *Satureja*. Those plants are rich in carvacrol and thymol, two isomers of phenolic “monoterpenes” that give the unique smell of these plants [31].

In the present study, we aimed to explore the COVID-19 severity of and the real-life use and possible effects of different medicinal plants on self-treated COVID-19 patients without previous vaccination in three different geographical areas in the Mediterranean basin, i.e., Italy, Lebanon, and Tunisia. In this context, this study explored the general use of medicinal plants in daily routines, focusing on their roles as preventive measures, and then specifically examined how participants used medicinal plants in response to the COVID-19 pandemic, investigating their applications for prevention, treatment, and symptom relief related to COVID-19.

## 2. Results

### 2.1. General Characteristics of the Enrolled Subjects and Clinical Presentation

The number of completed questionnaires was 812 and they were returned by 116 Italian subjects (14.3%), 557 Lebanese subjects (68.6%), and 139 Tunisian subjects (17.1%). Detailed information on the enrolled subjects according to the living area, sex, age, infection duration, and N/severity of COVID-19 symptoms are depicted in Table 1.

In the Italian cohort, 66.4% were females (*p* = 0.0001 vs. males). Overall, the mean age was 37.5 ± SEM 1.3 years. The mean duration of infection was 11.8 ± 0.6 days, the mean number of COVID-19 symptoms was 7.2 ± 0.3, with an overall symptom severity score of 17.9 ± 0.9, and no statistical difference between males and females.

In the Lebanese cohort, 58.2% were females (*p* = 0.02 vs. males). Overall, the mean age was 32.0 ± 0.6 years. The mean duration of infection was 14.7 ± 0.2 days, and the mean number of COVID-19 symptoms was 8.2 ± 0.3, with an overall symptom severity score of 23.9 ± 0.6. Females were significantly younger and reported more symptoms and higher severity scores than males (0.00003 < *p* < 0.0003).

In the Tunisian cohort, 68.3% were females (*p* = 0.0001 vs. males). Overall, the mean age was 33.0 ± 1.0 years. The mean duration of infection was 11.1 ± 0.5 days, and the mean number of COVID-19 symptoms was 10.3 ± 0.3, with an overall symptom severity score of 29.2 ± 1.2. Females were significantly younger than males (0.00003 < *p* < 0.0003).

**Figure 1:**
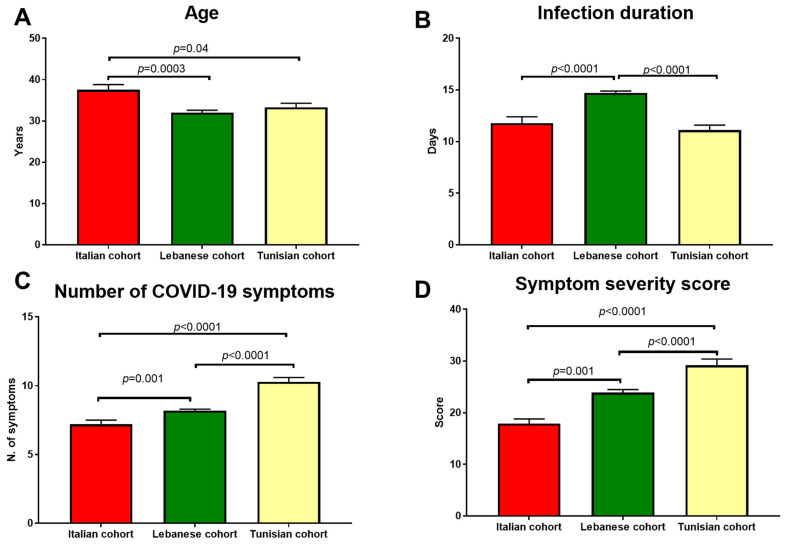
Comparison between the Italian, Lebanese, and Tunisian cohorts with respect to (**A**) age, (**B**) infection duration, (**C**) number of COVID-19 symptoms, and (**D**) the severity of COVID-19 symptoms. Significant differences were tested by ANOVA with post hoc tests.

The comparison between the Italian, Lebanese, and Tunisian cohorts with respect to age, infection duration, and N/severity of COVID-19 symptoms is depicted in Figure 1A–D. Italian participants were about five years older (*p* = 0.0006) than both Lebanese and Tunisian participants (Figure 1A). The duration of the COVID-19 infection was longer (*p* < 0.00001) in Lebanese subjects compared to Italian and Tunisian subjects (Figure 1B). The number and severity of symptoms increased progressively from the Italian to Lebanese to Tunisian cohorts (*p* < 0.00001) (Figure 1C,D).

### 2.2. Age Classes

Adult subjects (i.e., ≥18 years) represented the most patients in the three cohorts, i.e., 92.2% in Italy, 89.4% in Lebanon, and 93.5% in Tunisia. Details about the three age classes, i.e., pediatric, adult, and geriatric, are reported in Supplementary Table S1. Due to the scant number of cases in the pediatric and geriatric age groups in both the Italian and Tunisian cohorts, however, the most meaningful results belonged to the Lebanese cohort. Here, both the number and severity of COVID-19 symptoms were significantly less in pediatric participants (7.2%) compared to adults (0.001 ≤ *p* ≤ 0.001) but not the elderly.

### 2.3. Smoking

The classification of participating subjects according to cohorts and smoking status is depicted in Supplementary Table S2. In each cohort, the prevalence of smokers was significantly lower than non-smokers (0.0001 ≤ *p* ≤ 0.004). The number of smokers in the cohorts tended to increase from 30.2% in Italy to 36.3% in Tunisia and to 39.9% in Lebanon (*p* = n.s.). The percentage of smoking females was comparable between the cohorts. No differences existed between the infection duration and N/severity of symptoms between smokers and non-smokers in all cohorts.

### 2.4. Prevalence, Management, and Psychological Consequences of COVID-19 Symptoms and Post-COVID-19 Symptoms in the Three Cohorts

Table 2 compares the three cohorts according to the prevalence of the 13 COVID-19 symptoms, their management, psychological consequences, post-COVID-19 symptoms, and use of herbs.

The Tunisian cohort reported a consistently higher prevalence of almost all symptoms compared to the Italian and Lebanese cohorts, except for fatigue. In addition, fever and headache were comparable between the Tunisian and Italian cohorts, while loss of smell was comparable between the Tunisian and Lebanese cohorts. The three most recorded symptoms in the Italian cohort were headache (93.1%), fatigue (90.5%), and fever (84.5%); in the Lebanese cohort were fatigue (89.0%), headache (83.8%), and anosmia (80.3%); and in the Tunisian cohort were fatigue (94.2%), headache (94.2%), and fever (88.5%).

Most participants recorded a similar recourse to the doctor (Italy 80.2%, Lebanon 74.2%, and Tunisia 74.8%, *p* = n.s.) during the COVID-19 infection period. The percentage of participants who took medication in an attempt to decrease symptoms was significantly higher in the Tunisian cohort (89.9%) compared to the Italian (69.8%) and Lebanese (75.2%) cohorts. The percentage of hospitalized subjects was small and significantly lower in the Italian cohort (1.7%) than in the Lebanese (7.7%) and Tunisian (7.2%) cohorts. Aspects of the psychological consequences of COVID-19’s effects were reported by 25.9% in the Italian cohort, 30.9% in the Lebanese cohort, and 20.1% in the Tunisian cohort. Post-COVID-19 symptoms were significantly higher in Lebanese participants (48.8%) compared to Italian (22.4%) and Tunisian (31.7%) participants.

### 2.5. Use of Herbal Medicines

Table 3 shows that in general, the prevalence of regular (41–50%) and occasional (31–40%) use of herbs was comparable between the three cohorts. The prevalence of non-users decreased significantly across the Italian, Lebanese, and Tunisian cohorts. Notably, the prevalence of subjects believing that herbs can cure or prevent, and the prevalence of subjects using herbs for COVID-19 prevention and therapy, and those reporting effects on symptoms increased significantly from the Italian to the Lebanese to the Tunisian cohorts (0.00001 ≤ *p* ≤ 0.007).

The prevalence of specific herbs used for COVID-19 in the three cohorts is detailed in Table 3. Only 27 out of 116 (23.3%) of Italian subjects reported the use of herbs during COVID-19. The herbal tea, known in Italy as a “tisane’’, a mix of mainly *Matricaria chamomilla*, *Mentha piperita*, *Melissa officinalis*, and *Foeniculum vulgare*, was the most used herbal product (37.1%), followed by *Matricaria chamomilla* (22.2%) and *Camellia sinensis* (14.8%). A quarter of subjects (25.9%) reported the use of other herbs.

In the Lebanese cohort, 50.8% reported the use of herbs during COVID-19 infection. The popular Lebanese herbal tea known as “zhourat”, a mixture of mainly *Rosa damascen’*, *Matricaria chamomilla*, and *Micromeria* spp., was the most used herbal product (55.8%), followed by Za’atar plants (*Origanum syriacum*, *Thymbra spicata*, and *Satureja thymbra*) (12.0%), *Pimpinella anisum* (6.0%), and *Zingiber officinale* (5.7%). The other 12.7% of subjects reported the use of other herbs.

In the Tunisian cohort, 65.5% of subjects reported the use of herbs during COVID-19. Za’atar (*Thymbra capitata*) was the most used herbal product (52.7%), followed by *Verbena officinalis* (9.9%) and *Eucalyptus* sp. (6.6%). The other 19.8% of enrolled subjects reported the use of other herbs. The use of herbs was well tolerated and none of the subjects reported adverse effects, independently from the specific herb used.

### 2.6. Reported Effects of Herbal Medicine on COVID-19 Symptom Severity

The clinical presentation of COVID-19 in terms of the infection duration, severity of the 13 symptoms, number of symptoms, and severity of symptoms score, as well as COVID-19 management, were compared between subgroups of subjects taking the most employed herbal products and non-users of herbal products (Table 4).

In the Italian cohort, age and the duration of infection were comparable irrespective of herbal use. Data for symptoms showed variability mostly due to the scant number of observations since patients reported that, as compared to the no herb group, fever was increased in the tisane group (*p* = 0.01). Similarly, the management of the COVID-19 infection, including doctor visits and medication, displayed high variability with either increased or decreased effects in the “no herb” group and groups using herbs. In the Lebanese cohort, age and the duration of infection were comparable irrespective of herbal use. Regarding symptoms, no significant changes were observed between groups regarding diarrhea, fever, loss of smell, loss of taste, and vomiting. For abdominal pain, chest pain, cough, dyspnea, fatigue, headache, joint pain, and muscle pain, subjects in the zhourat group reported a significantly higher symptom severity compared to the control group, while subjects in the Za’atar group reported a significantly lower symptom severity (0.00005 ≤ *p* ≤ 0.02). The number of symptoms and symptom severity scores were significantly lower in the Za’atar group compared to the zhourat and no herb groups. In addition, the percentage of subjects who reported recourse to a doctor and medication use for COVID-19 were significantly lower in the “Za’atar” group compared to the other groups.

In the Tunisian cohort, age and the duration of infection were comparable irrespective of herbal use. Subjects in the tisane group reported a significantly higher severity of abdominal pain, cough, dyspnea, fatigue, headache, joint pain, and muscle pain compared to the no herb group (0.04 ≤ *p* ≤ 0.006). The N/symptoms were higher in the tisane group compared to the no herb group (0.02 ≤ *p* ≤ 0.002). Regarding management, a higher percentage of subjects in the Za’atar and tisane groups used recourse to the doctor (*p* < 0.05), while the number of subjects who took medications for COVID-19 were significantly higher in the tisane group compared to the no herb group (*p* < 0.05).

### 2.7. Emergence of Post-COVID-19 Symptoms and Possible Effects of Herbs

The emergence of post(long)-COVID-19 symptoms within 12 months in all subjects and according to the herb groups in the three cohorts are depicted in Table 5.

In the Italian cohort, 26 subjects (22.4%) reported post-COVID-19 symptoms. The most reported symptoms were headache (34.6%) and fatigue (19.2%). Gastrointestinal (GI) symptoms, including recurrent abdominal pain associated with changes in the frequency and shape of stool, were reported by 2 subjects (7.7%). The prevalence of post-COVID-19 symptoms was significantly lower in the no herb group (16.9%) compared to the tisane group (60.0%) and chamomile group (83.3%), with the results likely influenced by the limited number of cases in this group.

In the Lebanese cohort, 272 subjects (48.8%) reported post-COVID-19 symptoms. The most reported symptoms were fatigue (27.9%), headache (24.3%), and muscle pain (17.3%). The prevalence of post-COVID-19 was significantly lower in the Za’atar group (32.4%) compared to the zhourat (65.2%) and no herb groups (57.7%). Gastrointestinal symptoms were reported by 11 subjects (4.0%) and were comparable irrespective of herb use. In the Tunisian cohort, 44 subjects (31.7%) reported post-COVID-19 symptoms. The most reported post-COVID-19 symptoms were fatigue (25.0%) and headache (22.7%). The prevalence of post-COVID-19 was significantly lower in the Za’atar group (22.9%) compared to the tisane (55.6%) and no herb groups (58.3%). GI symptoms were reported by 2 subjects (4.5%).

## 3. Discussion

Generally, the use of medicinal plants in the Mediterranean area is popular and well-documented [11,33]. This is the first systematic study dealing with herbal use during the 1st wave of the COVID-19 pandemic in three different geographical areas in the Mediterranean basin, i.e., Italy, Lebanon, and Tunisia. We also extended the study to the post-COVID-19 period. In addition, the survey paves the way for several considerations, which include geographical, cultural, and scientific aspects.

In general, the three cohorts consisted of relatively young adults, and the Lebanese cohort was the most represented one. Some gender aspects emerged, since the number of females was higher than males in all countries, with a slightly younger age for females than males in Lebanon and Tunisia. The smoking status was comparable in both genders throughout the cohorts and did not influence the symptom characteristics. Whether the most prevalent manifestations of symptoms in the Tunisian cohort represent a true phenomenon remains to be investigated, although more participants used medications for COVID-19 than participants in the Italian and Lebanese cohorts.

The prevalence of COVID-19 symptoms was different between symptoms, and variability was recorded between cohorts. The highest prevalence was recorded for headache (83–94%), and the lowest prevalence was for vomiting (8–43%). Post-COVID-19 complications within 12 months after recovery (post(long)-COVID-19) were experienced by around one-quarter in the Italian cohorts, half in the Lebanese cohort, and one-third in the Tunisian cohort. These results are in line with other studies suggesting that the prevalence of post-COVID-19 syndrome varies widely between 8% and 70%, depending on the definition, living area, assessment method, and time points [34,35]. These complications were mainly in terms of fatigue, muscle pain, headache, and dyspnea. Here, we additionally observed an emergence of a few cases (7.7% in Italy, 4.0% in Lebanon, and 4.5% in Tunisia) of gastrointestinal symptoms, most likely associated with the diagnostic criteria of irritable bowel syndrome. The gastrointestinal involvement in post(long)- COVID-19 syndrome, its frequency, and its pathophysiology are still not completely understood. These results are in line with two recent international studies [19,36,37].

During the last few years, a considerable amount of population-based and ethnobotanical studies have documented the use of medicinal plants for COVID-19. These studies collectively explored the impact of the COVID-19 pandemic on herbal medicine practices across different regions, highlighting a range of medicinal plants traditionally used to manage COVID-19 symptoms. In the UK, practitioners adapted by prescribing plants like *Glycyrrhiza glabra* and *Echinacea* spp. for mild to moderate symptoms, with a noted need for consistent approaches in remote consultations [10]. Lithuania’s study raised concerns about the safe use of locally popular plants for respiratory health, especially when lacking formal approval or evidence-based guidelines [38]. In Morocco and Tanzania, ethnopharmacological research identified common medicinal plants like *Eucalyptus globulus* and *Azadirachta indica*, valued for their antiviral compounds, emphasizing the need for phytochemical validation [39,40]. Zimbabwe’s and Nigeria’s analyses highlighted the potential for integrating traditional African herbal knowledge with modern research to address COVID-19’s broader health impacts and explore phytochemicals with antiviral potential [41,42]. Together, these findings underscore the global interest in local herbal remedies as complementary or supportive treatments for COVID-19, with calls for systematic validation, safety evaluations, and culturally informed health governance.

Despite the possible socioeconomic and educational differences between the three cohorts, in Italy, the adherence to herbal medicine use was moderate. However, herbal remedies are deeply embedded in cultural practices in Lebanon and Tunisia, with different preferences for a broader range of herbs. This highlights how herbal remedies are not only influenced by the individual’s socioeconomic status or educational background but also by the rich local traditions and regional health practices prevalent in each country [43].

Such evident differences within the Mediterranean basin existed along with the increased use of a variety of medicinal plants for COVID-19, as reported in Table 3. In Italy, nearly one-quarter (23.3%) of participants used herbs for COVID-19 therapy, while half of the Lebanese participants (50.8%) reported herbal medicine use, and the majority (65.5%) of the Tunisian participants used herbs during COVID-19. In Italy, most herbal users reported herbal tea (tisane) as the most used, followed by chamomile and green tea. Tisane is a popular hot drink in Italy, which is usually made by steeping fresh or dried herbs in boiled water, and it can vary depending on the desired flavour and health benefits.

The Lebanese population uses medicinal plants as natural remedies against different disorders, including infective, acute, or chronic diseases. Indeed, in this study, half of the Lebanese participants (49%) reported the regular use of herbs, 34.2% occasional use, and most subjects (65%) agreed with the statement “Do you believe herbs can cure or prevent diseases?”. This confirms that the use of herbal medicine in Lebanon remains diffused and popular. Half of the Tunisian cohort (50%) reported the regular use of herbs, 40% occasional use, and most subjects (72%) agreed with the statement “Do you believe herbs can cure or prevent diseases?”.

In the Lebanese cohort, many patients relied on taking a group of herbs, which included zhourat, Za’atar, ginger, anise, green tea, chamomile, and other herbs, as they thought that taking them would heal them from the virus or at least alleviate the severity of the symptoms.

Za’atar is the most used herb since it is used in Tunisia as a hot drink or as a spicy herb for breakfast and dinner. Za’atar or *Thymbra capitata* L. is widely used in folk medicine as a stomachic, antispasmodic, or diaphoretic, and specifically against cough since it stimulates blood circulation [44].

The results of the current study revealed a distinct feature between different living areas regarding the use of herbs during the COVID-19 acute phase and the effects on COVID-19 and post-COVID-19 symptoms.

Our results apparently point to Za’atar as the most effective herbal product, in terms of beneficial effects on the COVID-19 symptom prevalence and severity in the Lebanese cohort. In fact, subjects in the Za’atar group reported lower overall symptom scores than subjects using no herbs or other herbal products. Interestingly, the prevalence of post-COVID-19 complications was significantly lower in the Za’atar group.

These effects might be explained, at least in part, by previous observations showing a broad spectrum of beneficial effects of Za’atar plants, such as an anti-inflammatory [45,46,47,48,49], antioxidant [50,51], antidiabetic [52,53,54], and hypolipidemic [51,55] agent. In detail, Za’atar plants such as *Origanum syriacum* L., *Thymbra spicata* L., and *Satureja thymbra* L. are perennial aromatic species belonging to the *Lamiaceae* family, and are popular as fresh or dried culinary herbs and food seasonings [56]. Essential oils of these plants are rich in volatile compounds, including thymol, p-cymene, and γ- terpinene, but most notably carvacrol [57]. Remarkably, those plants are also rich in phenolic compounds, including phenolic acids (rosmarinic acid) and flavonoids (both glycosides and aglycones) [58,59].

Studies on the effects of thyme-like plants and their primary compounds, carvacrol and thymol, suggest these components may offer antiviral, anti-inflammatory, and antioxidant benefits in the context of COVID-19 and SARS-CoV-2. *Thymus vulgaris* has traditionally been recognized for its antimicrobial and antiviral properties. Research shows that thyme and its bioactive compounds can suppress pro-inflammatory cytokines (e.g., TNF-α, and IL-6) and boost anti-inflammatory cytokines like IL-10, which may help manage inflammation linked to COVID-19 [60]. Carvacrol and thymol, two major constituents in thyme essential oil, have shown potential in inhibiting SARS-CoV-2 pathways. For example, in silico studies indicate that these compounds can bind to the catalytic domain of TMPRSS2 (a host enzyme facilitating viral entry), showing stability and potentially preventing SARS-CoV-2 from entering host cells [61]. Additionally, these compounds demonstrate binding affinities to the viral spike protein’s receptor-binding domain, potentially disrupting viral attachment and replication processes [62]. Further studies highlight that carvacrol and thymol can reduce oxidative stress and enhance antioxidant defenses, which may protect against virus-induced tissue damage [63]. Molecular docking analyses suggest that these compounds act as viral inhibitors by modulating immune responses and controlling CoV-induced lung inflammation, potentially making them valuable in alternative or complementary COVID-19 therapies [64]. A study on the ethanol and water extracts of thyme (*Thymus vulgaris*) showed its effects on the inhibition of the SARS-CoV-2 spike protein–ACE2 binding by 82.6 and 86.4%, respectively, which proved that thyme can be used to prevent SARS-CoV-2 infection and reduce the complications from the infection [41].

Carvacrol, one of the well-known bioactive compounds widely found in *Lamiaceae* plants, was proven to exhibit different beneficial effects [30]. A wide array of in vivo and in vitro studies have demonstrated the therapeutic potential and clinical significance of carvacrol as an anticancer [65], antibacterial [66,67], antioxidant [68], anti-inflammatory [69,70,71], and hepatoprotective [72] natural agent. A recent in vitro study on the inhibitory activity of *Origanum* essential oils and carvacrol against angiotensin-converting enzyme 2 (ACE2) and lipoxygenase (LOX), showed that carvacrol has 90.7% inhibitory activity against ACE2 in vitro [73]. In fact, many recent studies suggested that the selection of various FDA-approved antiviral compounds might yield promising results against COVID-19 infection. In this sense, Kumar et al. proved that CVL possesses a binding capacity to Mpro, a protease in the SARS-CoV-2 viral genome with a considerable role in the replication and maturation of the virus [74]. On the other hand, carvacrol exerts a potent suppressive activity against COX-2 expression and NFκB activation, and modulates the Nrf2/HO-1 signaling pathway, minimizing the acute inflammatory process and decreasing the release of some pro-inflammatory mediators such as IL-1β, TNF-α, and PGE2 [53,75], and it could down-regulate the activation of NF-κB signaling in lipopolysaccharide-treated macrophages [69]. Interestingly, different clinical studies showed that CVL consumption reduced the inflammatory status in pulmonary and respiratory diseases. In detail, CVL reduced serum pro-inflammatory cytokine and chemokine levels while increasing anti-inflammatory cytokine levels, improve respiratory symptoms [20], and reduced inflammatory cells and oxidant biomarkers, whereas it increased antioxidant biomarkers and improved pulmonary function [76]. In addition, in asthmatic patients, CVL increased pulmonary function tests, but respiratory symptoms, inflammatory cells, and hs-CRP levels were reduced [77,78].

Rosmarinic acid (RA), well-known as an “anti-inflammatory agent” [56], is present in high amounts in Za’atar plants [68]. RA possesses a significant activity as a radical scavenger in physiological environments [79] and has a wide range of pharmacological and biological activities, including antiviral, antibacterial, antioxidant, antimutagenic, and anti-inflammatory activities [53]. Many in vitro and in vivo studies have reported the anti-inflammatory effects of RA in inflammatory diseases such as asthma and respiratory diseases [80,81]. Recently, RA showed an antiviral effect against COVID-19 through a significant inhibition of the main protease (M^pro^) of SARS-CoV-2 [82].

However, these results were not observed in subjects who took Za’atar plants in the Tunisian cohort. This could be attributed to the huge variety of Za’atar plants that vary from the *Thymus*, *Origanum*, *Thymbra*, and *Satureja* plant families. People recognized Za’atar plants according to the culture and geographical area. Za’atar plants are known as the carvacrol- and thymol-containing plants. Those essential oils give the plants their favorable aroma and smell. Additionally, different plants of those families have different phytochemical compositions, especially in terms of the polyphenol content, which is mainly responsible for the possible antioxidant and anti-inflammatory properties. Za’atar in Lebanon mainly includes *Origanum syriacum* L. and *Thymbra spicata* L., with higher amounts of polyphenols that could reach 350 mg GAE/g of dry plant extract [83]. While in Tunisia, Za’atar plants include mainly *Thymbra capitata* L. and *thymus vulgaris* L., with a moderate polyphenol content ranging between 23 and 126 mg GAE/g dry extract [84,85].

Thus, the possible effects of Lebanese Za’atar plants on reducing COVID-19 symptom severity may be explained by their high number of polyphenols and essential oils with considerable antioxidant and anti-inflammatory effects. To give examples, Shen et al. revealed the anti-inflammatory role of *Origanum syriacum*, as it has the potential to decrease the LPS-induced iNOS and COX-2 enzyme levels [56]. *Thymbra spicata* was also shown to possess an antioxidant effect by decreasing ROS production, NO release, and lipid peroxidation [68,86].

Our study has some limitations. The sample size in our study was limited due to several factors. Our study was conducted during the first wave of the COVID-19 pandemic, prior to the availability of vaccines, when the number of individuals infected with the virus was relatively low. As a result, the sample size may not be fully representative of the broader population. We additionally recognize the importance of isolating the effects of plant use from other factors. While some overlap in symptom management might occur between herbal and allopathic treatments for similar conditions (such as influenza or pneumonia), our data collection specifically focused on the use of medicinal plants in general for COVID-19 prevention and for COVID-19 treatment. To ensure that the results reflected the actions of the plants to the best extent possible, we analyzed aspects related to the management of COVID-19. Our findings suggest that the users of Za’atar plants in Lebanon have a lower prevalence of conventional medication use and recourse to a doctor, which could strengthen our findings on the possible beneficial effects of Za’atar on COVID-19. Another limitation is that the cultural familiarity and daily use of plants and mixing or interaction of other plants in the Middle East and North Africa may influence perceptions of their efficacy. The cultural factor could impact the generalizability of our findings. Detailed explorations of how acute and regular exposure to these plants might shape health outcomes or perceived benefits in ways that may differ in populations without this background are needed.

Strongly emerging from this survey is the idea that at least in some geographical areas within the Mediterranean area, herbal medicines are considered options to tackle COVID-19 symptoms. This popular view stems from doubts about the effectiveness and toxicity of chemical drugs compared to the safety and efficiency of plants and plant-based drugs. Several herbal products have been clinically examined for COVID-19 treatment with promising results [16,87,88]. In this context, our study provides new information about the possible use of Za’atar plants or bioactive components as adjuvants for COVID-19 therapy.

## 4. Materials and Methods

### 4.1. Subjects

We conducted a web-based survey in three different free-living target populations in Italy, Lebanon, and Tunisia concerning the first wave of COVID-19 before vaccination in 2020 and throughout the 12 months after an acute COVID-19 infection. Participation was on a voluntary basis. The inclusion criteria encompassed individuals of all ages and genders who had been infected with SARS-CoV-2 at least once. We excluded participants with incomplete responses and those who had not undergone a PCR test.

The three cohorts consisted of 116 Italian, 557 Lebanese, and 139 Tunisian individuals. The protocol was approved by the local Ethics Committee at the Hospital Policlinico and University of Bari ‘Aldo Moro’ (study number 6558, protocol number 0085284).

### 4.2. Questionnaire

We used a tailored, anonymous, web-based “Google Form” questionnaire (Supplementary Text S1) designed in the English language and subsequently translated into Italian and Arabic (validated similarly to our previous study [89]). Sampling was conducted using random methods. We employed a simple and clear questionnaire to be distributed using social media (the link to the questionnaire was shared by the social media platforms WhatsApp, Email, and Facebook) or during face-to-face interviews with people without web access to have a simple random sample. This method was chosen to minimize bias and ensure that every individual in the target population had an equal chance of being selected, thereby supporting the study’s validity.

#### 4.2.1. COVID-19 Clinical Manifestations

The questionnaire consisted of 26 items investigating demographic characteristics, smoking status, and features of a COVID-19 infection, including the presence of 13 COVID-19-related symptoms (i.e., abdominal pain, chest pain, cough, diarrhea, dyspnea, fatigue, fever, headache, joint pain, loss of taste, loss of smell, muscle pain, and vomiting). The intensity of each symptom was scored using a semi-quantitative scale that ranges from 0 to 5, with each level representing a distinct degree of severity. Specifically, 0 indicates the absence of the symptom, 1 represents a very mild intensity, 2 corresponds to mild, 3 to moderate, 4 to high, and 5 signifies the maximal intensity. This type of scale is commonly used in clinical research to assess symptom severity across various conditions. A symptom score for each participant was therefore calculated by the sum of each score (maximal possible score 65). We included a section regarding the management of COVID-19, including allopathic treatments (i.e., use of conventional medications), recourse to the doctor, or hospitalization. The following aspects were also evaluated: psychological consequences (i.e., appearance of at least one of the following: stress, anxiety, and fear), and major post-COVID-19 symptoms, namely, fatigue, muscle pain, headache, dyspnea, and others. Besides a few gastrointestinal symptoms during COVID-19, we investigated specific post-COVID-19 gastrointestinal (GI) symptoms, including recurrent abdominal pain associated with changes in the frequency and form of stool, using the Rome IV Diagnostic Questionnaire for Functional Gastrointestinal Disorders in Adults (R4DQ) [90].

#### 4.2.2. Use of Medicinal Plants

The methodology used in this section consisted of a structured survey aimed at assessing both the general and COVID-19-specific use of medicinal herbs among participants. The survey was administered to capture self-reported data, allowing participants to describe their habits and beliefs in their own words, where applicable. The mixed response types (multiple choice and open ended) allowed for both a quantitative analysis (e.g., frequency of use and beliefs about efficacy) and qualitative insights (e.g., types of herbs, preparation methods, and symptom relief). Quantitative responses were analyzed to determine usage patterns and belief prevalence, while qualitative data provided deeper insights into individual practices and perceptions.

To establish baseline information on general usage, participants were asked if they used medicinal herbs (e.g., in the form of teas, infusions, or other natural products) with the options to select “Yes”, “No”, or “Once in a while”. This question sought to gauge the frequency of regular herb usage in daily life. Their beliefs regarding the effectiveness of medicinal herbs for general health purposes were specifically assessed by asking if they thought herbs could cure or prevent diseases. The responses available were “Yes”, “No”, and “I don’t know” to capture not only beliefs but also any uncertainty regarding herbal efficacy.

To explore the application of medicinal herbs specifically for COVID-19, the survey included targeted questions. Participants were asked if they used medicinal herbs as a preventive measure before infection, with “Yes” or “No” responses, to understand if herbs were used proactively. During the COVID-19 infection phase, participants were questioned on whether they used medicinal herbs, also with a “Yes” or “No” response. This was followed by a series of questions if they responded “Yes”: type of herbs used—participants could specify which herbs they used through a short-answer response, allowing for detailed data on specific plants or combinations. In addition, the survey provided options for regular use (one or more times daily), occasional use (once every 2–3 days), or rare use (once a week) to determine usage patterns and intensity during the infection. An open-ended question asked participants to describe the method of preparation (e.g., herb infusion in water or use as a food ingredient) and the quantity consumed to capture detailed information on herbal administration methods and doses. Finally, participants were asked whether they believed the herbs alleviated their symptoms (“Yes”, “No”, or “I don’t know”) to assess perceived outcomes. If participants reported symptom relief, they were asked to specify which symptoms were alleviated through a short-answer response. This question aimed to link specific symptoms with potential herbal benefits.

This methodology enabled a thorough understanding of medicinal herb use, both in general and within the specific context of the COVID-19 pandemic. The structured questions ensured comparability, while open-ended responses provided detailed insights, which were particularly valuable for identifying traditional or region-specific practices.

### 4.3. Statistical Analysis

Data are expressed as the means and standard errors (SEMs) for continuous variables or as proportions and percentages for categorical variables. The chi-square test (proportions), the *t*-test (unpaired data), and the Kruskal–Wallis multiple comparison Z-value test were employed to evaluate intra- or inter-group differences. All statistical analyses were performed using NCSS software (NCSS LLC, Kaysville, UT, USA), and statistical significance was declared if a two-sided *p*-value was <0.05 [91].

## 5. Conclusions

This study provides valuable insights into the use of herbal medicine, particularly polyphenolic-rich thyme-like plants, during the COVID-19 pandemic across three Mediterranean regions: Italy, Lebanon, and Tunisia. The use of herbal remedies varies significantly across the Italian, Lebanese, and Tunisian cohorts, reflecting cultural preferences and local plant availability. In Italy, herbal remedy use is relatively low (23.3%), with a preference for mixed herbal teas containing chamomile (*Matricaria chamomilla*), peppermint (*Mentha piperita*), and lavender (*Lavandula angustifolia*). In contrast, Lebanon shows higher usage (50.8%) and a strong preference for “zhourat”, a blend of herbs like damask rose (*Rosa damascena*), *Chamomile*, linden flowers (*Tilia cordata*), and Za’atar plants (*Origanum*, *Thymbra*, and *Satureja* plants). Tunisia has the highest proportion of users (65.5%), with Za’atar (*Thymbra capitata*) as the predominant choice, alongside other herbs like verveine (*Verbena officinalis*) and *Eucalyptus*. These differences highlight the role of regional traditions and the local flora in shaping herbal remedy practices across cultures. In Lebanon, the use of Za’atar, a thyme-like plant, was particularly prominent and correlated with a reduction in COVID-19 symptoms. This suggests that the polyphenolic compounds present in Za’atar, such as thymol and carvacrol, may offer protective effects against COVID-19. Despite the widespread use of herbal medicine, there remains a lack of robust scientific evidence supporting their efficacy in treating COVID-19. This study highlights the need for further research to explore the therapeutic potential of these herbal compounds. Clinical trials and in-depth studies are essential to validate the observed benefits and understand the mechanisms through which these plants may exert their effects.

## Figures and Tables

**Table 1:** Demographic characteristics of the 812 participants according to gender, age, infection duration, number of symptoms, and symptom severity score in the three cohorts.

	Total	Males	Females	*p* (M vs. F)
ITALIAN COHORT				
N (%)	116 (100%)	39 (33.6%)	77 (66.4%)	0.0001
Age (years)	37.5 ± 1.3	39.4 ± 2.5	36.5 ± 1.5	0.29
Infection duration (days)	11.8 ± 0.6	11.4 ± 0.7	11.9 ± 0.8	0.64
N of COVID-19 symptoms	7.2 ± 0.3	7.3 ± 0.5	7.2 ± 0.3	0.94
Symptom severity score	17.9 ± 0.9	17.9 ± 1.7	17.9 ± 1.2	0.97
LEBANESE COHORT				
N (%)	557 (100%)	233 (41.8%)	324 (58.2%)	0.02
Age (years)	32.0 ± 0.6	34.7 ± 1.0	30.0 ± 0.7	0.00008
Infection duration (days)	14.7 ± 0.2	14.9 ± 0.3	14.5 ± 0.3	0.41
N of COVID-19 symptoms	8.2 ± 0.1	7.6 ± 0.2	8.6 ± 0.2	0.00003
Symptom severity score	23.9 ± 0.6	21.2 ± 0.9	25.9 ± 0.7	0.0003
TUNISIAN COHORT				
N (%)	139	44 (31.7%)	95 (68.3%)	0.0001
Age (years)	33.3 ± 1.0	37.3 ± 2.0	31.5 ± 1.2	0.009
Infection duration (days)	11.1 ± 0.5	10.7 ± 0.5	11.8 ± 1.0	0.27
N of COVID-19 symptoms	10.3 ± 0.3	9.8 ± 0.5	10.5 ± 0.3	0.22
Symptom severity score	29.2 ± 1.2	25.7 ± 1.8	30.8 ± 1.6	0.054

Data are expressed as means ± SEMs. N: number of subjects. The difference between genders was tested by a *t*-test. M = males; F = females.

**Table 2:** Prevalence, management, and psychological consequences of COVID-19 symptoms, post-COVID-19 symptoms, and use of herbs in the three cohorts.

	ITALIAN COHORT (N = 116)	LEBANESE COHORT (N = 557)	TUNISIAN COHORT (N = 139)	*p* ITA vs. LEB	*p* ITA vs. TUN	*p* LEB vs. TUN
**COVID-19 Symptoms**						
Abdominal pain *	48 (41.4%)	245 (44.0%)	89 (64.0%)	0.60	<0.00001	<0.00001
Chest pain	46 (39.7%)	288 (51.7%)	100 (71.9%)	0.02	<0.00001	<0.00001
Cough	91 (78.4%)	431 (77.4%)	127 (91.4%)	0.80	0.003	0.0002
Diarrhea *	19 (16.4%)	212 (38.1%)	78 (56.1%)	<0.00001	<0.00001	<0.00001
Dyspnea	60 (51.7%)	291 (52.2%)	105 (75.5%)	0.92	0.0001	<0.00001
Fatigue	105 (90.5%)	496 (89.0%)	131 (94.2%)	0.64	0.25	0.06
Fever	98 (84.5%)	362 (65.0%)	123 (88.5%)	<0.00001	0.19	<0.00001
Headache	108 (93.1%)	467 (83.8%)	131 (94.2%)	0.01	0.53	0.0007
Joint pain	73 (62.9%)	414 (74.3%)	122 (87.8%)	0.01	<0.00001	0.0008
Loss of taste	51 (44.0%)	381 (68.4%)	121 (87.1%)	<0.00001	<0.00001	<0.00001
Loss of smell	47 (40.5%)	447 (80.3%)	118 (84.9%)	<0.00001	<0.00001	0.1
Muscle pain	85 (73.3%)	414 (74.3%)	120 (86.3%)	0.81	0.009	0.003
Vomiting *	10 (8.6%)	115 (20.6%)	60 (43.2%)	0.002	<0.00001	<0.00001
**Management of symptoms**						
Recourse to a doctor	93 (80.2%)	413 (74.2%)	104 (74.8%)	0.17	0.3	0.87
Medication for COVID-19	81 (69.8%)	419 (75.2%)	125 (89.9%)	0.22	<0.00001	0.0002
Hospitalization	2 (1.7%)	43 (7.7%)	10 (7.2%)	0.02	0.04	0.83
Psychological consequences	30 (25.9%)	172 (30.9%)	28 (20.1%)	0.28	0.27	0.01
Post-COVID-19 symptoms	26 (22.4%)	272 (48.8%)	44 (31.7%)	<0.00001	0.1	0.0003
**General use of herbs**						
Regular	48 (41.4%)	274 (49.2%)	70 (50.4%)	0.12	0.15	0.80
Occasional	36 (31.0%)	190 (34.1%)	55 (39.6%)	0.52	0.16	0.23
Non-user	32 (27.6%)	93 (16.7%)	14 (10.0%)	0.006	0.0003	0.053
Believe that the use of herbs can cure or prevent	40 (34.7%)	336 (64.1%)	101 (72.4%)	<0.00001	<0.00001	0.007
**Use of herbs for COVID-19**						
For prevention	3 (2.6%)	168 (30.2%)	82 (59.0%)	<0.00001	<0.00001	<0.00001
For therapy	27 (23.3%)	283 (50.8%)	91 (65.5%)	<0.00001	<0.00001	0.0002
Effects on symptoms	17 (14.7%)	219 (39.3%)	100 (71.9%)	<0.00001	<0.00001	<0.00001

Data are expressed as numbers and percentages (%). Differences between cohorts were tested by the chi-square test. The asterisk (*) indicates gastrointestinal symptoms.

**Table 3:** Use of specific medicinal plants for COVID-19 infection in the three cohorts.

ITALIAN COHORT (N = 116)	Common Name	Scientific Name	N (%)
**Non-users**			89 (76.7%)
**Users**			27 (23.3%)
**Plants used**	Herbal tea (tisane)	Mixture of *Matricaria chamomilla*, *Mentha piperita*, *Melissa officinalis*, *Foeniculum vulgare*, *Rosmarinus officinalis*, *Lavandula angustifolia*, and *Thymus vulgaris*	10/27 (37.1%)
	Chamomile	*Matricaria chamomilla*	6/27 (22.2%)
	Green tea	*Camellia sinensis*	4/27 (14.8%)
	Others		7/27 (25.9%)
LEBANESE COHORT (N = 557)			N. (%)
Non-users			274 (49.2%)
Users			283 (50.8%)
**Plants used**	Herbal tea (zhourat)	Mixture of mainly *Rosa damascen’*, *Matricaria chamomilla*, *Micromeria* sp., and *Crataegus monogyna*	158/283 (55.8%)
	Za’atar	*Origanum syriacum*, *Thymbra spicata*, and *Satureja thymbra*	34/283 (12.0%)
	Anise	*Pimpinella anisum*	17/283 (6.0%)
	Ginger	*Zingiber officinale*	16/283 (5.7%)
	Green tea	*Camellia sinensis*	13/283 (4.6%)
	Chamomile	*Matricaria chamomilla*	9/283 (3.2%)
	Others		36/283 (12.7%)
TUNISIAN COHORT (N = 139)			N. (%)
**Non-users**			48 (34.5%)
**Users**			91 (65.5%)
**Plants used**	Za’atar	*Thymbra capitata*	48/91 (52.7%)
	Tisane (verveine)	*Verbena officinalis*	9/91 (9.9%)
	Eucalyptus	*Eucalyptus* sp.	6/91 (6.6%)
	Cloves	*Syzygium aromaticum*	5/91 (5.5%)
	Herbal tea	*Camellia sinensis, Rosmarinus officinalis,* and *Mentha piperita*	5/91 (5.5%)
	Others		18/91 (19.8%)

Data are expressed as numbers and percentages, N (%).

**Table 4:** Symptoms reported during the COVID-19 infection according to the use of herbs in the three cohorts.

ITALIAN COHORT (N = 116)	No Herbs (N = 89)	Tisane (N = 10)	Chamomile (N = 6)	*p*
Age	38.4 ± 1.6	35.8 ± 3.3	33.8 ± 4.4	0.90
Infection duration	11.6 ± 0.6	10.6 ± 1.7	14.3 ± 1.5	0.11
***Symptoms***				
Abdominal pain	1.0 ± 0.1	0.3 ± 0.2	1.3 ± 0.6	0.22
Chest pain	1.0 ± 0.1	0.4 ± 0.2	0.5 ± 0.3	0.46
Cough	1.9 ± 0.2	2.9 ± 0.4	2.0 ± 0.3	0.15
Diarrhea	0.2 ± 0.1	0.2 ± 0.1	0.3 ± 0.2	0.71
Dyspnea	1.1 ± 0.1	0.7 ± 0.3	0.5 ± 0.5	0.23
Fatigue	2.5 ± 0.2	2.1 ± 0.5	2.5 ± 0.4	0.79
Fever	2.1 ± 0.2 *	3.5 ± 0.6 *	3.2 ± 0.3	0.01
Headache	2.7 ± 0.2	2.4 ± 0.3	2.3 ± 0.5	0.67
Joint pain	1.5 ± 0.2	0.4 ± 0.2	1.3 ± 0.5	0.05
Loss of smell	1.1 ± 0.2	1.0 ± 0.4	1.0 ± 0.4	0.88
Loss of taste	1.0 ± 0.2	1.1 ± 0.4	1.0 ± 0.4	0.83
Muscle pain	1.9 ± 0.2	1.7 ± 0.4	2.3 ± 0.3	0.71
Vomiting	0.2 ± 0.1	0.2 ± 0.2	0.0 ± 0.0	0.93
N of symptoms	7.2 ± 0.3	6.8 ± 0.7	8.5 ± 0.7	0.48
Symptom score	18.1 ± 1.1	16.9 ± 2.0	18.3 ± 3.0	0.90
***Management***				
Recourse to doctor	78 (87.7%) *#	5 (50.0%) *	3 (50.0%) #	<0.05
Medication for COVID-19	59 (66.3%)	7 (70.0%)	6 (100%)	n.s.
Hospitalization	2 (2.3%)	0 (0.0%)	0 (0.0%)	n.a.
LEBANESE COHORT (N = 557)	No herbs (N = 274)	Zhourat (N = 158)	Za’atar (N = 34)	*p*
Age	31.2 ± 0.8	31.5 ± 1.0	33.8 ± 3.0	0.82
Infection duration	14.8 ± 0.3	15.1 ± 0.5	13.3 ± 0.5	0.10
***Symptoms***				
Abdominal pain	1.0 ± 0.1 *	1.3 ± 0.1 *	0.3 ± 0.1 *	0.0008
Chest pain	1.3 ± 0.1 *	1.7 ± 0.1 *	0.5 ± 0.2 *	0.0001
Cough	1.9 ± 0.1 *	2.4 ± 0.1 *#	1.5 ± 0.2 #	0.0005
Dyspnea	1.4 ± 0.1 *	1.5 ± 0.1 #	0.5 ± 0.2 *#	0.003
Diarrhea	1.0 ± 0.1	1.0 ± 0.1	0.4 ± 0.1	0.06
Fatigue	2.9 ± 0.1 *	3.2 ± 0.1 *	2.3 ± 0.2 *	0.001
Fever	1.8 ± 0.1	1.7 ± 0.1	1.1 ± 0.2	0.15
Headache	2.4 ± 0.1 *	2.8 ± 0.1 *#	2.1 ± 0.3 #	0.02
Joint pain	2.3 ± 0.1 *	2.7 ± 0.1 *	1.2 ± 0.3 *	0.00004
Loss of smell	2.6 ± 0.1	2.5 ± 0.1	1.9 ± 0.3	0.19
Loss of taste	2.2 ± 0.1	2.0 ± 0.1	1.8 ± 0.3	0.67
Muscle pain	2.2 ± 0.1 *	2.6 ± 0.1 *	1.3 ± 0.3 *	0.0002
Vomiting	0.6 ± 0.1	0.6 ± 0.1	0.0 ± 0.0	0.15
N of symptoms	8.0 ± 0.2 *	8.9 ± 0.2 *	6.3 ± 0.5 *	0.0007
Symptom score	23.6 ± 0.8 *	26.1 ± 1.0 #	14.9 ± 2.2 *#	0.00005
***Management***				
Recourse to doctor	206 (75.2%) *	124 (78.5%) #	20 (58.8%) *#	<0.05
Medication for COVID-19	197 (71.9%) *	138 (87.3%) #	18 (52.9%) *#	<0.05
Hospitalization	26 (9.5%) *	12 (7.6%)	1 (2.9%) *	<0.05
TUNISIAN COHORT (N = 139)	No herbs (N = 48)	Za’atar (N = 48)	Tisane (N = 9)	*p*
Age	30.3 ± 1.2	35.2 ± 2.3	26.3 ± 2.4	0.15
Infection duration	11.0 ± 1.0	11.5 ± 0.3	11.0 ± 1.3	0.18
***Symptoms***				
Abdominal pain	1.4 ± 0.2 *	1.8 ± 0.2	2.6 ± 0.5 *	0.04
Chest pain	2.0 ± 0.2	1.9 ± 0.2	3.0 ± 0.6	0.15
Cough	2.2 ± 0.2 *#	2.8 ± 0.2 *	3.4 ± 0.2 #	0.01
Diarrhea	1.3 ± 0.2	1.6 ± 0.2	1.4 ± 0.4	0.31
Dyspnea	1.6 ± 0.2 *#	2.4 ± 0.1 *	2.8 ± 0.5 #	0.01
Fatigue	2.9 ± 0.2 *	2.7 ± 0.2 #	4.1 ± 0.3 *#	0.02
Fever	2.4 ± 0.2	2.9 ± 0.2	3.6 ± 0.3	0.07
Headache	2.8 ± 0.2 *	2.5 ± 0.2 #	4.1 ± 0.3 *#	0.01
Joint pain	2.5 ± 0.3 *	2.4 ± 0.2 #	4.1 ± 0.3 *#	0.006
Loss of smell	2.3 ± 0.3	2.4 ± 0.2	3.7 ± 0.6	0.06
Loss of taste	2.3 ± 0.3 *	2.4 ± 0.2 #	3.9 ± 0.4 *#	0.02
Muscle pain	2.4 ± 0.3 *	2.4 ± 0.2 #	3.8 ± 0.4 *#	0.048
Vomiting	0.6 ± 0.2	1.2 ± 0.2	0.9 ± 0.5	0.052
N of symptoms	9.0 ± 0.6 *	11.6 ± 0.3 *	11.3 ± 0.7	0.002
Symptom score	26.6 ± 2.4 *	29.4 ± 1.6 #	41.3 ± 4.5 *#	0.02
***Management***				
Recourse to doctor	30 (62.6) *#	43 (89.6%) *	8 (88.9%) #	<0.05
Medication for COVID-19	39 (81.3%) *	47 (97.9%) *	8 (88.9%)	<0.05
Hospitalization	4 (8.3%)	4 (8.3%)	0 (0.0%)	n.a

Age, infection duration, and the 13 symptoms are expressed as averages ± SEMs derived from a 0–5 semiquantitative scale. Management is expressed as numbers and percentages (N, %). Differences between percentages were tested by the chi-square test; differences between averages were tested by the Kruskal–Wallis multiple comparison Z-value test. N, number of subjects. n.s., Not significant; n.a.; not applicable. Similar symbols (*, #) indicate a significant difference (*p* < 0.05).

**Table 5:** Post-COVID-19 symptoms according to the use of herbs in the three cohorts.

**ITALIAN COHORT (N = 116)**	**Total (N = 116)**	**No Herbs (N = 89)**	**Tisane (N = 10)**	**Chamomile (N = 6)**
N of subjects (%)	26 (22.4%)	15 (16.9%) *#	6 (60.0%) *	5 (83.3%) #
Fatigue	5 (19.2%)	3 (20.0%)	1 (16.7%)	1 (16.7%)
Muscle pain	3 (11.5%)	1 (6.7%)	1 (10.0%)	1 (16.7%)
Headache	9 (34.6%)	5 (33.3%)	2 (20.0%)	2 (33.3%)
Dyspnea	3 (11.5%)	2 (13.3%)	0 (0.0%)	1(16.7%)
Gastrointestinal symptoms	2 (7.7%)	2 (13.3%)	0 (0.0%)	0 (0.0%)
Others	4 (15.4%)	2 (13.3%)	2 (20.0%)	0 (0.0%)
**LEBANESE COHORT (N = 557)**	**Total (N = 557)**	**No herbs (N = 274)**	**Zhourat (N = 158)**	**Zaatar (N = 34)**
N of subjects (%)	272 (48.8%)	158 (57.7%) *	103 (65.2%) #	11 (32.4%) *#
Fatigue	76 (27.9%)	34 (21.5%)	35 (34.0%)	4 (36.7%)
Muscle pain	47(17.3%)	27 (17.1%)	17 (16.5%)	1 (9.1%)
Headache	66 (24.3%)	43 (27.2%)	26 (25.2%)	5 (45.5%)
Dyspnea	45 (16.5%)	25 (15.8%)	18 (17.5%)	0 (0.0%)
Gastrointestinal symptoms	11 (4.0%)	9 (5.7%)	2 (1.9%)	0 (0.0%)
Others	27 (9.9%)	20 (12.7%)	5 (4.9%)	1 (9.1%)
**TUNISIAN COHORT (N = 139)**	**Total (N = 139)**	**No herbs (N = 48)**	**Zaatar (N = 48)**	**Tisane (N = 9)**
N of subjects (%)	44 (31.7%)	28 (58.3%) *	11 (22.9%) *#	5 (55.6%) #
Fatigue	11 (25.0%)	7 (25.0%)	2 (18.2%)	1 (20.0%)
Muscle pain	5 (11.4%)	3 (10.7%)	2 (18.2%)	0 (11.1%)
Headache	10 (22.7%)	6 (21.4%)	4 (36.4%)	1 (20.0%)
Dyspnea	5 (11.4%)	3 (10.7%)	0 (0.0%)	1 (20.0%)
Gastrointestinal symptoms	2 (4.5%)	1 (3.6%)	2 (18.2%)	0 (0.0%)
Others	11 (25.0%)	8 (28.6%)	1(9.1%)	2 (40.0%)

Data are expressed as numbers and percentages; N (%). Difference between percentages tested by chi-square test. Similar symbols (*, #) indicate a significant difference (*p* < 0.05). GI, gastrointestinal; N, Number of subjects.

## Data Availability

The raw data supporting the conclusions of this article will be made available by the authors on request.

## Supplementary Materials

The following supporting information can be downloaded at https://www.mdpi.com/article/10.3390/plants13233340/s1, Supplementary Text S1; Table S1: Classification of participating subjects according to cohorts and age classes. Table S2: Classification of participating subjects according to cohorts and smoking status
