# Supplementary material for: Herbal Medicine in Three Different Mediterranean Living Areas During the COVID-19 Pandemic: The Role of Polyphenolic-Rich Thyme-like Plants
Source: Plants (Basel). 2024 Nov 28;13(23):3340. doi: 10.3390/plants13233340 (PMC11644039; doi:10.3390/plants13233340)
Supplement: Supplementary file 1 [file plants-13-03340-s001.zip › plants-3272689-supplementary.pdf]

SUPPLEMENTARY MATERIAL

Supplementary Text S1

Italian Questionnaire

"Clinica Medica Augusto Murri" (Responsabile P. Portincasa)
Informazioni sull'infezione da coronavirus: sintomi, complicanze e uso di piante medicinali nei pazienti COVID-19
La compilazione e' anonima ed i dati saranno trattati con la più assoluta confidenzialita'. Si ringrazia per la collaborazione.
L'Unità Operativa di Medicina Interna "Clinica Medica Augusto Murri" (Responsabile P. Portincasa) comprende diversi ambulatori (Medicina interna, gastroenterologia, epatologia, cardiologia, endocrinologia, malattie rare e metaboliche) e svolge diversi protocolli di ricerca clinici e molecolari. La clinica si avvale della collaborazione di diverse figure professionali: medici, ricercatori, dottori di ricerca, specializzandi, infermieri e studenti con ampia collaborazione internazionale. Le nostre competenze cliniche e di ricerca comprendono: studi epidemiologici, questionari, sviluppo di alimenti funzionali, sindrome post-COVID-19, studi sulla percezione e gradevolezza degli alimenti e sulla motilità gastrointestinale, studi della funzione fisiologica dell'asse intestino-fegato tramite ultrasonografia e breath test, valutazione di disturbi metabolici (obesità, diabete, fegato grasso, ecc..), malattie rare, e medicina di genere.

Se ha avuto il COVID-19 prima della vaccinazione, per favore risponda alle domande di seguito circa la prima infezione COVID-19

Eta
Short answer text
Sesso

Maschio
Femmina

Email
Short answer text
Citta'
Short answer text
Fumi?
Si
No

Che lavoro fai?
Short answer text
Sapevi dell'esistenza del Coronavirus prima che si diffondesse nel decembre 2019?
Si
No

Quanti giorni è durata l'infezione da Coronavirus? (Fino a quando non appare un test PCR negativo)
Short answer text

Durante l'infezione da Coronavirus:

Description (optional)

Sei stato in quarantena domiciliare?
Si
No

Hai consultato un medico?
Si
No

Hai dovuto prendere farmaci per alleviare i sintomi?
Si
No

Se sì, quale farmaco e per quanti giorni?
Short answer text
Sei stato ricoverato in ospedale?
Si
No

Se la tua risposta è sì,dove?:
Degenza
Terapia intensiva

Se sì, quanti giorni hai soggiornato in ospedale?
Short answer text
Hai avuto bisogno di respirazione artificiale?
Si
No

Quali sono i tuoi sintomi?
0 (nessuno)
1 (mai grave)
2 (non grave)
3 (Intermedio)
4 (grave)
5 (Molto grave)
Febbre
Diarrea
Vomito
Mal di testa
Perdita dell'olfatto
Perdita del gusto
Respiro corto
Dolore al torace
Dolore addominale

Stanchezza
Tosse
Insonnia
Dolore articolari
Mialgia (dolore muscolare)
Sei stato psicologicamente influenzato dall'infezione da Coronavirus?
Si
No

Se sì, quale effetto psicologico hai riscontrato?
Short answer text
Dopo l'infezione entro 12 mesi, hai avuto complicazioni?
Si
No

Se sì, quali sono state le complicazioni?
Short answer text

Hai sofferto di sindrome dell'intestino irritabile o dolori addominali ricorrenti (almeno una volta alla settimana) negli ultimi 3 mesi?
Si
No

Se la tua risposta è sì, quali sono i sintomi?
Short answer text

Sintomi legati alla defecazione
Si
No

Cambiamento nella frequenza della defecazione
Si
No

Cambiamento nella forma (aspetto) delle feci
Si
No

Usi le erbe medicinali in generale? (Tè, Tisane, Prodotti naturali...)
Si
No
Una volta ogni tanto

Pensi che le erbe medicinali aiutino a guarire o prevenire le malattie? (In generale)
Si
No
Non lo so

Le erbe medicinali sono state utilizzate per prevenire il Coronavirus prima dell'infezione?
Si
No

Hai usato erbe medicinali durante il periodo di infezione da Coronavirus?
Si
No

Se sì, quali sono state le erbe medicinali che ha usato?
Short answer text
Se sì, quante volte hai usato le erbe?
regolarmente (una o più volte al giorno)
occasionalmente (una volta ogni 2-3 giorni)
Raramente (una volta alla settimana)

Se sì, descrivi brevemente il metodo di preparazione e la quantità (infusione erbe con acqua, aggiunte come ingrediente al cibo ecc..)
Short answer text
Secondo te, le erbe medicinali hanno aiutato ad alleviare i sintomi?
Si
No
Non lo so

Se sì, quali sintomi hai alleviato usando le erbe medicinali?
Short answer text
Feedback/Suggerimenti
Short answer text

In caso di persistenza di eventuali sintomi post-COVID, o di eventuali malattie metaboliche (Obesità, diabete, fegato grasso, disturbi gastrointestinali ecc..) potete contattarci ai seguenti recapiti: Email: ricerca.clinicamedicamurri@gmail.com, Tel: +39-080-5592731

Oppure lasciate il vostro recapito (Tel, email) e sarete ricontattati

Short answer text

Supplementary Text - Lebanese and Tunisian Questionnaires

معلومات حول الإصابة بفايروس كورونا :العوارض ,المضاعفات,واستخدام الأعشاب الطبية لدى مرضى كوفيد -19

اذا كنت قد اصبت بفايروس كوفيد-١٩

قبل ان تتلقى

اي جرعة من اللقاح حتى الآن، يرجى أن تملأ الإستمارة أدناه

عن أول اصابة

كوفيد-١٩

العمر

*

Short answer text

الجنس

*

الدولة، العنوان

*

Short answer text

هل كنت تعرف بوجود فايروس كورونا من قبل انتشاره في تشرين الثاني من العام 2019؟

*

(سلبي pcr لغاية ظهور فحص) كم كانت مدة الإصابة؟

*

Short answer text

خلال الإصابة:

Description (optional)

هل كنت في الحجر المنزلي؟

*

هل استشرت طبيب؟

*

هل اضررت لأخذ أدوية لتخفيف العوارض؟

*

هل دخلت المستشفى؟

*

..إذا كان جوابك نعم ,هل أحتجت

إذا كان جوابك نعم ,كم يوم بقيت في المستشفى؟

Short answer text

هل احتجت تنفس اصطناعي؟

*

ما هي العوارض التي أصابتك؟

*

(لا شيئ) 0

(ليس شديد أبداً) 1

(ليس شديد) 2

(متوسط) 3

(شديد) 4

(شديد جداً) 5

ارتفاع الحرارة

سعال جاف

تعب و انحطاط

ألم في العضل

ألم في المفاصل

اسهال

تقيء
ألم في الرأس
فقدان حاسة الشم
فقدان حاسة التذوق
ضيق في التنفس
ألم في الصدر
ألم في المعدة
هل تأثرت نفسياً بإصابة؟
*

إذا كان جوابك نعم ,ما هو الأثر النفسي الذي اختبرته؟
Long answer text
؟ خلال 12 شهر بعد الإصابة ,هل عانيت من مضاعفات
*

إذا كان جوابك نعم ,ما هي المضاعفات؟
Long answer text
هل عانيت من عوارض القولون العصبي او آلام البطن المتكررة (على الأقل مرة في الأسبوع)في آخر ٣ اشهر؟

اذا كان جوابك نعم، ما هي العوارض؟
Short answer text

عوارض متعلقة بالتغوط
*

تغير عدد مرات التغوط
*

تغيير في شكل (مظهر)البراز
*

هل تستعمل الأعشاب الطبية بشكل عام؟)شاي,زهورات(...
*

هل تعتقد بأن الأعشاب الطبية تساعد في الشفاء أو الوقاية من الأمراض؟
*

هل استعملت الأعشاب الطبية للوقاية من فايروس كورونا قبل الإصابة؟
*

إذا كان جوابك نعم ,ما هي الأعشاب؟
Long answer text
هل استعملت الأعشاب الطبية خلال فترة الإصابة بالفايروس؟
*

إذا كان جوابك نعم ,ما هي الأعشاب؟
Long answer text
برأيك ,هل ساعدت الأعشاب الطبية في تخفيف حدة الأعراض؟
إذا كان جوابك نعم ,ما هي الأعراض التي خفت بتناول الأعشاب؟
Long answer text
ملاحظات/ اقتراحات
Long answer text

Supplementary Table S1. Classification of participating subjects according to cohorts and age classes.

	Pediatric (<18y)	Adults (18-64y)	Elderly (≥65y)	P
**ITALIAN COHORT (N=116)**	2 (1.7%)	107 (92.2%)	7 (6.1%)	
Females	1 (50%)	74 (69.2%)	2 (28.6%)	
Age (years)	17.0±0.0	35.8±1.1	69.0±1.4	n.a
Infection duration (days)	11.0±4.0	11.7±0.6	12.0±1.3	n.a.
N. of COVID-19 symptoms	10.5±0.5	7.1±0.3	7.6±1.2	n.a.
Symptom severity score	40.0±9.0	17.3±0.9	20.9±4.4	n.a.
**LEBANESE COHORT (N=557)**	40 (7.2%)	498 (89.4%)	19 (3.4%)	
Females	24 (60%)	294 (59.0%)	6 (31.6%)	
Age (years)	12.3±0.8*	32.1±0.5*	69.1±1.0*	<0.0001
Infection duration (days)	15.2±1.1	14.6±0.2	15.6±1.2	0.51
N. of COVID-19 symptoms	6.3±0.6*	8.4±0.1*	7.3±0.7	0.001
Symptom severity score	14.9±2.0*	24.8±0.6*	20.6±3.6	0.00001
**TUNISIAN COHORT (N=139)**	7 (5.1%)	130 (93.5%)	2 (1.4%)	
Females	5 (71.4%)	88 (67.7%)	2 (100%)	
Age (years)	13.4±1.7	33.8±1.0	70.0±5.0	<0.0001
Infection duration (days)	13.3±0.7	10.9±0.5	11.0±1.0	n.a.
N. of COVID-19 symptoms	10.9±0.5	10.2±0.3	13.0±0.0	n.a.
Symptom severity score	26.4±3.2	29.1±1.3	40.5±11.5	n.a

Data are expressed as mean ± SEM. Difference between groups tested by ANOVA and post-hoc test. Similar asterisks indicate significant differences between groups. n.a., not appliable

Supplementary Table S2. Classification of participating subjects according to cohorts and smoking status.

	Smokers	Non-smokers	P
**ITALIAN COHORT (N=116)**	**35 (30.2%)**	**81 (69.8%)**	0.00001
Females	21 (60.0%)<br>Smoking females 18.1%	56 (69.1%)	
Age (years)	41.2±2.7	35.9±1.4	0.06
Infection duration (days)	12.8±1.4	11.3±0.5	0.60
N. of COVID-19 symptoms	7.3±0.5	7.2±0.3	0.85
Symptom severity score	18.8±1.9	17.5±1.1	0.42
**LEBANESE COHORT (N=557)**	**222 (39.9%)**	**335 (60.1%)**	0.004
Females	112 (50.5%)<br>Smoking females 20.1%	212 (63.3%)	
Age (years)	31.9±0.9	32.0±0.8	0.9
Infection duration (days)	14.6±0.3	14.8±0.3	0.74
N. of COVID-19 symptoms	8.0±0.2	8.3±0.2	0.20
Symptom severity score	23.1±0.9	24.5±0.7	0.20
**TUNISIAN COHORT (N=139)**	**37 (36.3%)**	**102 (63.7%)**	0.0001
Females	26 (70.3%)<br>Smoking females 18.7%	69 (67.6%)	
Age (years)	33.3±1.8	33.3±1.3	1.0
Infection duration (days)	11.1±0.9	11.0±0.5	0.84
N. of COVID-19 symptoms	10.4±0.5	10.2±0.3	0.80
Symptom severity score	31.7±2.5	28.2±1.4	0.24

Data are expressed as mean ± SEM. N: number of subjects. Difference between gender tested by T-test.
